# Overexpression of *GmAKT2* potassium channel enhances resistance to soybean mosaic virus

**DOI:** 10.1186/1471-2229-14-154

**Published:** 2014-06-03

**Authors:** Lian Zhou, Hongli He, Ruifang Liu, Qiang Han, Huixia Shou, Bao Liu

**Affiliations:** 1 State Key Laboratory of Plant Physiology and Biochemistry, College of Life Sciences, Zhejiang University, Hangzhou 310058, P. R. China; 2 Key Laboratory of Molecular Epigenetics of Ministry of Education, Northeast Normal University, Changchun 130024, China

**Keywords:** Soybean mosaic virus, Resistance, Potassium channel, *GmAKT2*

## Abstract

**Background:**

Soybean mosaic virus (SMV) is the most prevalent viral disease in many soybean production areas. Due to a large number of SMV resistant loci and alleles, SMV strains and the rapid evolution in avirulence/effector genes, traditional breeding for SMV resistance is complex. Genetic engineering is an effective alternative method for improving SMV resistance in soybean. Potassium (K^+^) is the most abundant inorganic solute in plant cells, and is involved in plant responses to abiotic and biotic stresses. Studies have shown that altering the level of K^+^ status can reduce the spread of the viral diseases. Thus K^+^ transporters are putative candidates to target for soybean virus resistance.

**Results:**

The addition of K^+^ fertilizer significantly reduced SMV incidence. Analysis of K^+^ channel gene expression indicated that *GmAKT2,* the ortholog of *Arabidopsis* K^+^ weak channel encoding gene *AKT2*, was significantly induced by SMV inoculation in the SMV highly-resistant genotype Rsmv1, but not in the susceptible genotype Ssmv1. Transgenic soybean plants overexpressing *GmAKT2* were produced and verified by Southern blot and RT-PCR analysis. Analysis of K^+^ concentrations on different leaves of both the transgenic and the wildtype (Williams 82) plants revealed that overexpression of *GmAKT2* significantly increased K^+^ concentrations in young leaves of plants. In contrast, K^+^ concentrations in the old leaves of the *GmAKT2*-Oe plants were significantly lower than those in WT plants. These results indicated that *GmAKT2* acted as a K^+^ transporter and affected the distribution of K^+^ in soybean plants. Starting from 14 days after inoculation (DAI) of SMV G7, severe mosaic symptoms were observed on the WT leaves. In contrast, the *GmAKT2*-Oe plants showed no symptom of SMV infection. At 14 and 28 DAI, the amount of SMV RNA in WT plants increased 200- and 260- fold relative to *GmAKT2*-Oe plants at each time point. Thus, SMV development was significantly retarded in *GmAKT2*-overexpressing transgenic soybean plants.

**Conclusions:**

Overexpression of *GmAKT2* significantly enhanced SMV resistance in transgenic soybean. Thus, alteration of K^+^ transporter expression is a novel molecular approach for enhancing SMV resistance in soybean.

## Background

Soybean (*Glycine max* (L.) Merr.), a major source of protein and oil in the human diet is an important crop world wide. Soybean mosaic virus (SMV) is the most prevalent viral disease in many soybean production areas [1]. Infection with SMV causes severe symptoms, including mosaic symptoms (light and dark green areas, chlorosis, and leaf curl), necrosis (necrotic areas, stem browning, and stem-tip necrosis), and seed mottling, resulting in serious yield losses [2]. Yield losses due to SMV infection range from 8% to 50% under natural field conditions [3], to total crop loss during severe outbreaks [4].

There are seven SMV strain groups (G1-G7) and three resistance loci (*Rsv1*, *Rsv3*, and *Rsv4*) reported in soybean [5-8]. Soybean germplasm with *Rsv1* locus are resistant to SMV strain groups G1-G3, but susceptible to strains G5-G7 [9]. In contrast, lines containing *Rsv3* confer resistance to strain groups G5-G7, and condition stem-tip necrosis and/or mosaic symptoms to G1-G4 [10]. The *Rsv4* locus was reported to produce seedling resistance to most SMV isolates but systemic symptoms can appear as plants mature [11]. *Rsv1, 3,* and *4* loci were mapped to chromosome 13, 14 and 2, respectively [11-15] and candidate genes for loci *Rsv1* and *Rsv3* have been putatively identified. *Rsv1* was shown to link to a cluster of six non-Toll interleukin 1 receptor (TIR) nucleotide-binding site leucine rich repeat (NBS-LRR) genes [16], and recently directly verified using virus-induced gene silencing approach [17]. Furthermore, *Rsv3* gene has been reported to associate with a cluster of the coiled-coil nucleotide-binding leucine-rich repeat (CC-NB-LRR) resistance genes [18]. Overall, due to the large number of *Rsv* loci and alleles, multiple SMV strains, and the rapid evolution in avirulence/effector genes under R gene selective pressure, breeding to pyramid these *Rsv* loci is complicated.

Alternative modes of resistance to pathogen resistance are also possible. Potassium (K^+^), the most abundant inorganic solute in plant cells, plays many important regulatory roles in plant development and stress responses [19]. High K^+^ status decreases the occurrence of many diseases [20]. Perrenoud (1990) reviewed more than 2,000 studies and found a correlation between K^+^ status and disease incidence [21]. A high K^+^ status reduced bacterial, fungal, and viral diseases in 69%, 70%, and 41% of the studies, respectively. In seventeen case studies on viral disease, high K^+^ status reduced the incidence of viral diseases in nine studies, although five studies showed the opposite effect [22]. The correlation between K^+^ status of plants and their susceptibility to pathogens involves changes in their primary metabolite profiles and distribution and the hormonal pathways in plants with altered K^+^ status [20]. K^+^ status affects the function of multiple plant enzymes, and thus it changes metabolite profiles and concentrations [23]. The changes in metabolites ultimately alter the susceptibility of plants to pathogens. Furthermore, K^+^ status also affects plant hormonal pathways, i.e. salicylic acid (SA) and jasmonic acid (JA) pathways [20], that are involved in hypersensitive responses or aquired systemic resistance to pathogens.

Plants absorb and transport K ions using a number of transport proteins [24]. The superfamily of voltage-gated K^+^ channels, the *Shaker* family, plays roles in K^+^ uptake and K^+^ loading into xylem and phloem [25-28]. K channel proteins mediate either K^+^ uptake (inward-rectifying K^+^ channels, K_in_) or K^+^ release (outward-rectifying K^+^ channels, K_out_) [29]. The *Arabidopsis* K^+^ weak channel *AKT2* can mediate both K^+^ uptake and release [30,31]. In *Arabidopsis*, *AKT2* is predominantly expressed in phloem tissues, guard cells, and root stellar tissues [32,33]. *AKT2* regulates transport of K^+^ and other small molecules in phloem through its roles in electric cell signaling and membrane excitability [28]. *AKT2* may be involved in plant stress responses by adjusting potassium gradients that are important energy sources in plant vascular tissues [28,32-34].

We observed that the incidence of SMV can be significantly reduced by application of K^+^ fertilizer. Analysis of K^+^ channel gene expression in SMV-resistant and SMV-susceptible cultivars showed that the expression of *GmAKT2*, the soybean inner K^+^ transporter gene, was induced in the resistant variety, but not in the susceptible cultivar. Overexpression of *GmAKT2* significantly increased SMV resistance in the SMV susceptible cultivar Williams 82. Our results suggest that alterlation of the expression of the K^+^ transporter *AKT2* is a novel molecular approach to genetically enhance SMV resistance in soybean.

## Results

### The effect of K^+^ supply on SMV incidence

**Figure 1:**
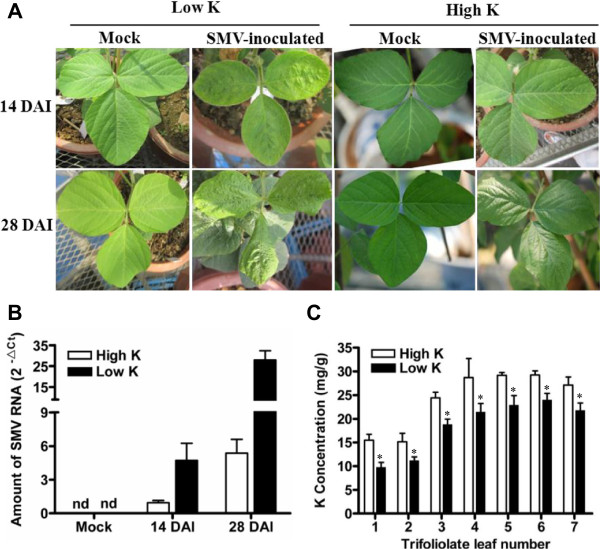
**SMV resistance and K**^**+ **^**concentrations of Williams 82 soybean plants grown under different K supplies. (A)** SMV symptoms at 14 or 28 days after inoculation (DAI) with SMV in soybean plants grown in soil pots with low (36.5 mg/kg) or high (200 mg/kg) levels of K^+^. Unrolled unifoliate leaves of 10-day-old soybean plants were mechanically inoculated with SMV strain G7 or buffer (Mock). Photos were taken on the newest leaf of the plants, which were 2^nd^ trifoliate leaf at 14 DAI and 7^th^ trifoliate leaf at 28 DAI, respectively. **(B)** Amount of SMV RNA detected by quantitative RT-PCR (qRT-PCR). The middle leaflets of the leaves of Williams 82 plants which grown in low or high K soil were sampled at 14 and 28 DAI to extract total RNA for qRT-PCR analysis of SMV. Transcript levels were calculated using the formula 2^-ΔCt^ for the expression levels relative to *GmACTIN*. **(C)** K^+^ concentrations in individual leaves of plants grown in either high- or low-K soil. The first through seventh trifoliate leaves from 45-day-old soybean plants were sampled. Data represent means of three biological replicates with error bars indicating SD. Asterisks indicate a significant difference between high- and low-K soil (*P < 0.05).

To investigate whether K^+^ supply affected the resistance of soybean plants to SMV, the susceptible soybean cultivar Williams 82 was planted in pots containing low-K^+^ soil with or without the addition of K^+^ fertilizer. Ten-day-old seedlings with completely unrolled unifoliate leaves in both K^+^-sufficient and -starvation treatments were inoculated with SMV-G7 or buffer (Mock). At 14 DAI, mild mottling and crinkled appearances were observed in the second unrolled trifoliate leaves in plants grown in low-K^+^ soil (Figure 1A). In contrast, only slight chlorotic spots were observed in the second unrolled trifoliate leaves of plants grown in high-K^+^ soil (Figure 1A). At 28 DAI, all SMV-infected soybean plants grown in K^+^-starvation soil showed typical SMV-susceptible symptoms: the young trifoliate leaves were severely mosaic and curled (Figure 1A). However, plants grown in high-K^+^ soil showed late-susceptible symptoms or resistant phenotypes (Figure 1A).

To quantify the amount of SMV viral accumulation, quantitative RT-PCR (qRT-PCR) was carried out to determine the amount of SMV RNA in these soybean plants. As shown in Figure 1B, SMV RNA can be detected in all inoculated plants at 14 and 28 DAI, indicating that the inoculation process was effective. The amount of SMV RNA in soybean plants grown in K^+^-starvation soil was significantly higher than that in K^+^-sufficient soil at both 14 and 28 DAI (Figure 1B), suggesting that sufficient supply of K^+^ could reduce SMV accumulation.

Leaf K^+^ concentrations from the first to the seventh trifoliate leaves of 45-day-old plants grown in high- or low-K^+^ soil were measured. As expected, K^+^ concentrations in older leaves were significantly lower than in younger leaves. K^+^ concentrations in the leaves of plants with sufficient K^+^ supplies were significantly higher than in the corresponding leaves of those in K^+^-starvation soil (Figure 1C).

### Differential expression of *GmAKT2* in SMV-resistant and -susceptible genotypes in response to SMV infection

**Figure 2:**
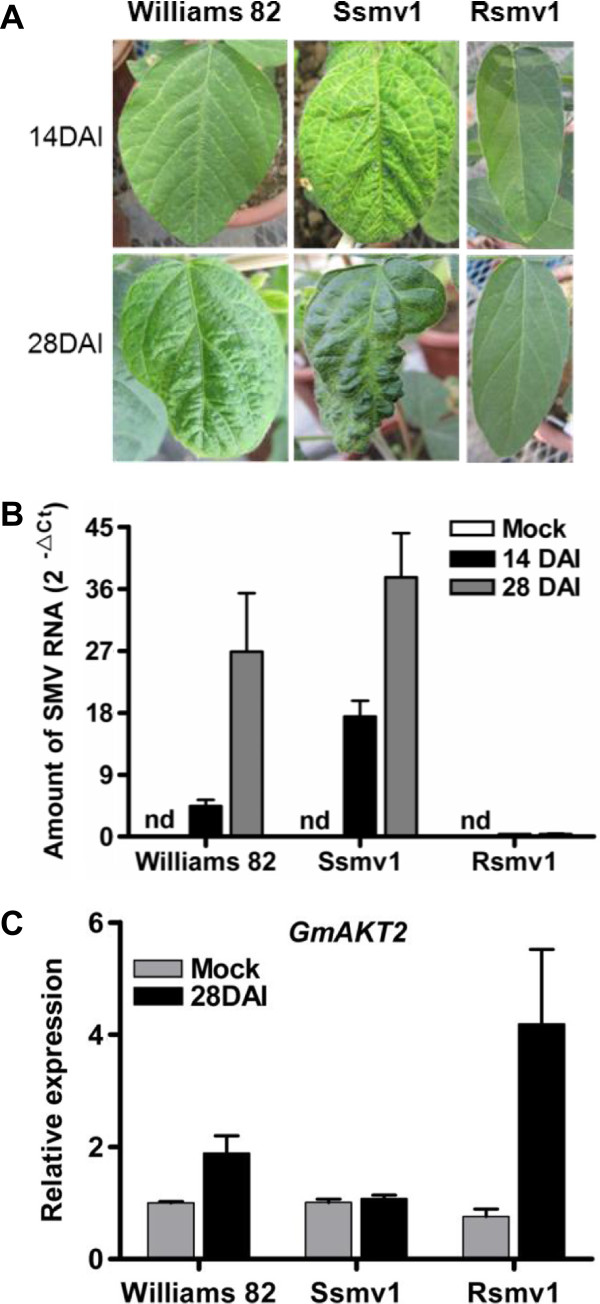
**SMV resistance and *GmAKT2 *expression in three soybean genotypes. (A)** Symptoms of three soybean genotypes at 14 and 28 DAI. Rsmv1, SMV highly-resistant; Williams 82, susceptible; Ssmv1, highly-susceptible. Ten-day-old soybean plants in low-K soil with unrolled unifoliate leaves were mechanically inoculated with SMV G7 or buffer (Mock). **(B)** Amount of SMV RNA detected by qRT-PCR. Leaves of three soybean genotypes were sampled at 14 and 28 DAI to extract total RNA for qRT-PCR analysis. Transcript levels were calculated using the formula 2^-ΔCt^ for the expression levels relative to *GmACTIN*. Data represent means of three biological replicates with error bars indicating SD. **(C)** Relative *GmAKT2* expression in three soybean genotypes at 28 DAI. Leaves of three soybean genotypes were sampled at 28 DAI to extract total RNA for qRT-PCR analysis. Transcript levels were calculated using the formula 2^-ΔΔCt^ for the expression levels relative to *GmACTIN*. Data represent means of three biological replicates with error bars indicating SD.

To determine whether K^+^ transporter genes were involved in soybean responses to SMV infection, the transcript levels of the soybean K^+^ channel genes *GmAKT1* and *GmAKT2* in response to SMV infection were investigated. Genotypes used for the assay included Rsmv1, Williams 82, and Ssmv1, which are highly-resistant, susceptible, and highly susceptible to SMV, respectively (Figure 2A). The amounts of SMV RNA accumulation detected by qRT-PCR matched to the symptoms of viral infection (Figure 2B). While the inoculation of SMV G7 significantly increased the accumulation of SMV RNA in Williams 82 and Ssmv1 plants at 14 and 28 DAI, but not in Rsmv1 plants. Compared to Williams 82, Ssmv1 plant was more susceptible to SMV infection (Figure 2B).

To determine whether SMV infection will affect the expression of K^+^ transporters, qRT-PCR was performed to examine *GmAKT1* and *GmAKT2* expression in first trifoliate leaves at 28 DAI. *GmAKT2* transcript levels were significantly induced by SMV infection in Rsmv1, but not in Ssmv1 (Figure 2C). In Williams 82, *GmAKT2* transcript levels were induced to a lower extent than in Rsmv1 (Figure 2C). In contrast, *GmAKT1* expression was not affected by SMV inoculation in all three genotypes (data not shown).

### *GmAKT2* was preferentially expressed in aerial tissues and induced by K^+^ starvation

**Figure 3:**
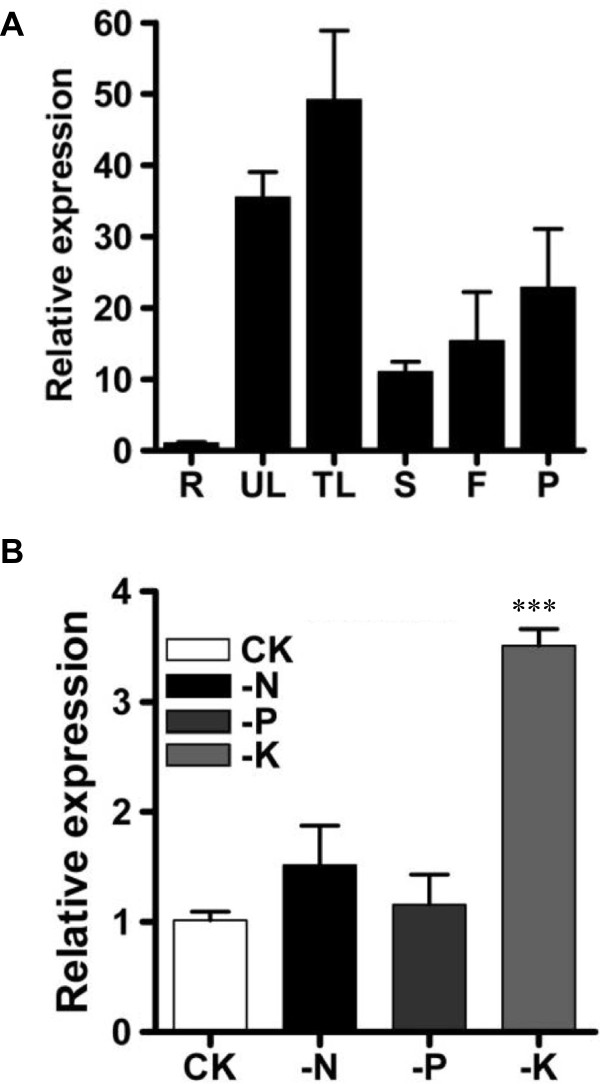
***GmAKT2 *expression in different tissues and leaves of Williams 82 under various nutrient supply conditions. (A)** Relative *GmAKT2* expression in roots (R), unifoliate leaves (UL), trifoliate leaves (TL), stems (S), flowers (F), and pods (P). Soybean seedlings were grown hydroponically in growth chambers for 6 weeks. **(B)** Relative *GmAKT2* expression in leaves of plants grown under different nutrient stress. Ten-day-old soybean seedlings were transferred into modified half-strength Hoagland hydroponic solution (CK) or solutions lacking nitrogen (-N), phosphate (-P), or potassium (-K) for 7 days. Transcript levels were calculated using the formula 2^-ΔΔCt^ for the expression levels relative to *GmACTIN*. Data represent means of three biological replicates with error bars indicating SD. Asterisks indicate a significant difference between the control and treated samples (***P < 0.001).

To analyze *GmAKT2* expression in various soybean tissues, qRT-PCR was performed on RNA samples extracted from 6-week-old Williams 82 seedlings grown hydroponically. *GmAKT2* was preferentially expressed in aerial tissues, especially leaves (Figure 3A). To determine whether the expression of *GmAKT2* was affected by the status of nutrient supplies, 10-day-old soybean seedlings were transferred to solution cultures lacking nitrogen, phosphate, or potassium. *GmAKT2* expression was specifically induced by K^+^ deficiency (Figure 3B).

### Generation of transgenic soybean overexpressing *GmAKT2*

**Figure 4:**
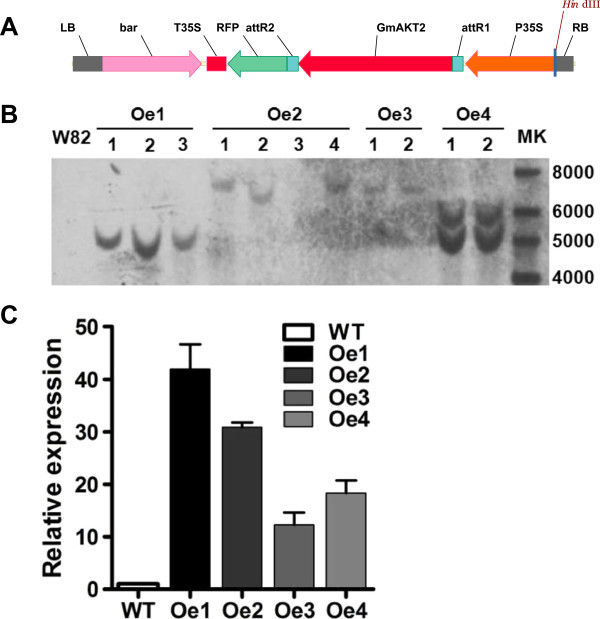
**Construction and verification of *GmAKT2*-overexpressing transgenic soybean plants. (A)** T-DNA region of the *GmAKT2* overexpression vector. LB, left border; RB, right border; *bar*, phosphinothricin acetyl transferase gene; P35S, CaMV double 35S promoter; T35S, CaMV 35S terminator; RFP, red fluorescence protein gene. **(B)** Southern blot analysis of *GmAKT2*-overexpressing transgenic lines. Oe1, Oe2, Oe3, and Oe4 represent four independent *GmAKT2* overexpressing lines. Genomic DNA of 2-week-old T_1_ transgenic seedlings and the non-transformed recipient soybean genotype Williams 82 was extracted and digested with *Hind*III. *bar* gene was digoxigenin labeled and used as the probe for analysis. **(C)** Relative *GmAKT2* expression in the leaves of the transgenic lines. T_2_ generations of Oe1, Oe2, Oe3, and Oe4 and WT were cultured in a hydroponic system. RNA was extracted from the leaves of 2-week-old seedlings. *GmAKT2* transcript levels were determined by qRT-PCR. Data represent means of three biological replicates with error bars indicating SD. *GmACTIN* expression was used as the internal control.

The *GmAKT2* full-length cDNA was amplified from Rsmv1, Williams 82, and Ssmv1, respectively. Sequence analysis showed that there is no variation on the sequence of *GmAKT2* cDNA (Additional file 1: Figure S1). To assay the effect of *GmAKT2* on SMV resistance, the obtained *GmAKT2* cDNA driven by a modified CaMV 35S promoter (Figure 4A) was introduced into Williams 82 via *Agrobacterium*-mediated transformation. Four independent T_1_ transgenic lines overexpressing *GmAKT2* (*GmAKT2*-Oe1, 2, 3, and 4) were verified by Southern blot analysis using the phosphinothricin acetyl transferase gene (*bar*) as the probe. Oe1, 2, 3, and 4 were four independent transgenic lines each containing a single copy (Oe1, 2, and 3) or two copies (Oe4) of the transgene (Figure 4B). qRT-PCR analysis of the T_2_ transgenic soybean plants showed that the transgene *GmAKT2* was highly expressed in all transgenic lines (Figure 4C). The two transgenic events, Oe1 and Oe2, showing the highest levels of *GmAKT2* gene expression, were selected for further experiments.

The growth of transgenic soybean plants was compared with the growth of recipient soybean plants. No significant differences were observed between wild type and transgenic plants in the agronomic traits investigated, including plant height, stem diameter, branch number, node number, pod number, seed number, seed yield, and 100-seed weights (Table 1).

**Table 1:** **Agronomic performance of Williams 82 (WT) and *GmAKT2*-overexpressing transgenic (Oe1 and Oe2) plants in field condition**

**Genotype**	**WT**	**Oe1**	**Oe2**
Plant height (cm)	61.5 ± 6.5^a^	62.1 ± 9.9^a^	66.9 ± 7.0^a^
Stem diameter (cm)	0.71 ± 0.22^a^	0.75 ± 0.22^a^	0.78 ± 0.13^a^
Branch number	6.3 ± 0.5^a^	6.7 ± 1.0^a^	6.3 ± 0.9^a^
Node number	19.3 ± 0.5^a^	19.4 ± 0.5^a^	21.5 ± 0.5^a^
Pod number/plant	126.5 ± 37.0^a^	132.3 ± 26.5^a^	120.5 ± 27.9^a^
Seed number/plant	241.4 ± 52.9^a^	256.9 ± 68.6^a^	236.1 ± 43.3^a^
Seed weight (g)/plant	30.1 ± 9.8^a^	29.9 ± 9.7^a^	29.8 ± 6.4^a^
100 seed weight (g)	11.9 ± 2.2^a^	11.7 ± 1.3^a^	12.8 ± 2.0^a^

Data collected from a field experiment in Anhui province, 2012. Data are given as means ± SD. Different letters in a row indicate significant differences (LSD, P <0.05).

### *GmAKT2* overexpression altered K distribution in soybean leaves

**Figure 5:**
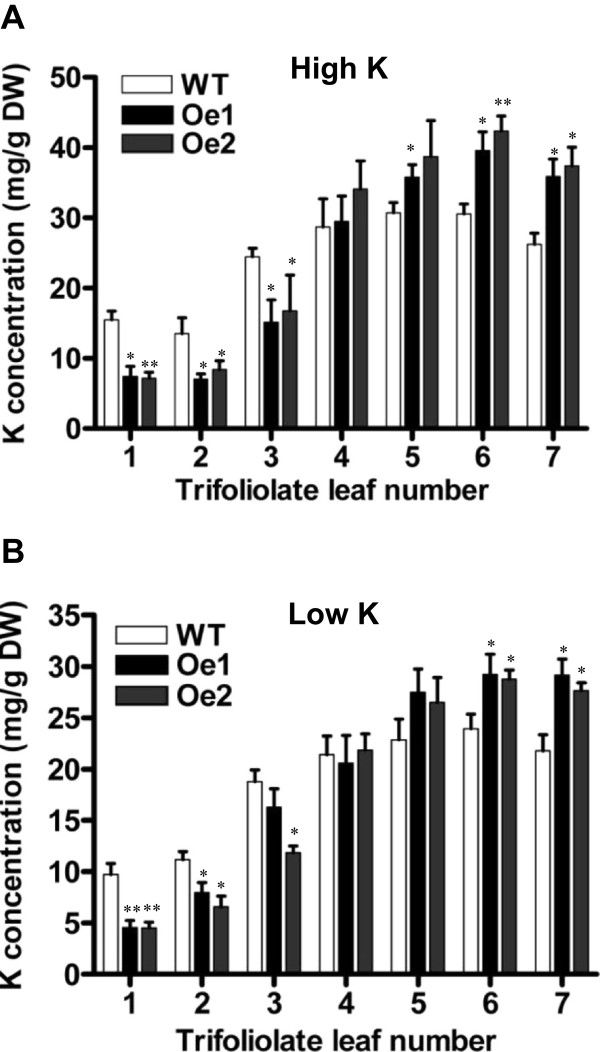
**Potassium concentrations in the leaves of WT and transgenic soybean plants grown in high- or low-K soil.** WT and transgenic lines were germinated and grown in pots with soil containing high K (**A**, 200 mg/kg) or low K (**B**, 36.5 mg/kg) for 6 weeks. The first through seventh trifoliate leaves from each treatment were collected and K^+^ contents were measured. Data represent means of four biological replicates with error bars indicating SD. Asterisks indicate a significant difference between the WT and transgenic lines (*P < 0.05; **P < 0.01).

K^+^ concentrations in the first to seventh trifoliate leaves of 6-week-old transgenic and WT plants grown in K^+^-sufficient or -starvation soil were analyzed. Compared to WT plants, overexpression of *GmAKT2* significantly increased K^+^ concentrations in young leaves (fifth through seventh trifoliate leaves) of plants grown in K^+^-sufficient or -starvation conditions (Figure 5A, 5B). For instance, K^+^ concentrations in the seventh leaves of the *GmAKT2*-overexpression lines were 27-40 % higher than that of the WT plants (Figure 5A, 5B). In contrast, K^+^ concentrations in the old leaves (first through third trifoliate leaves) of the *GmAKT2*-Oe plants were significantly lower than those in the WT plants (Figure 5A, 5B). These results indicated that *GmAKT2* acted as a K^+^ transporter and affected the distribution of K^+^ in soybean plants.

### *GmAKT2* overexpression increased SMV resistance in transgenic soybean

**Figure 6:**
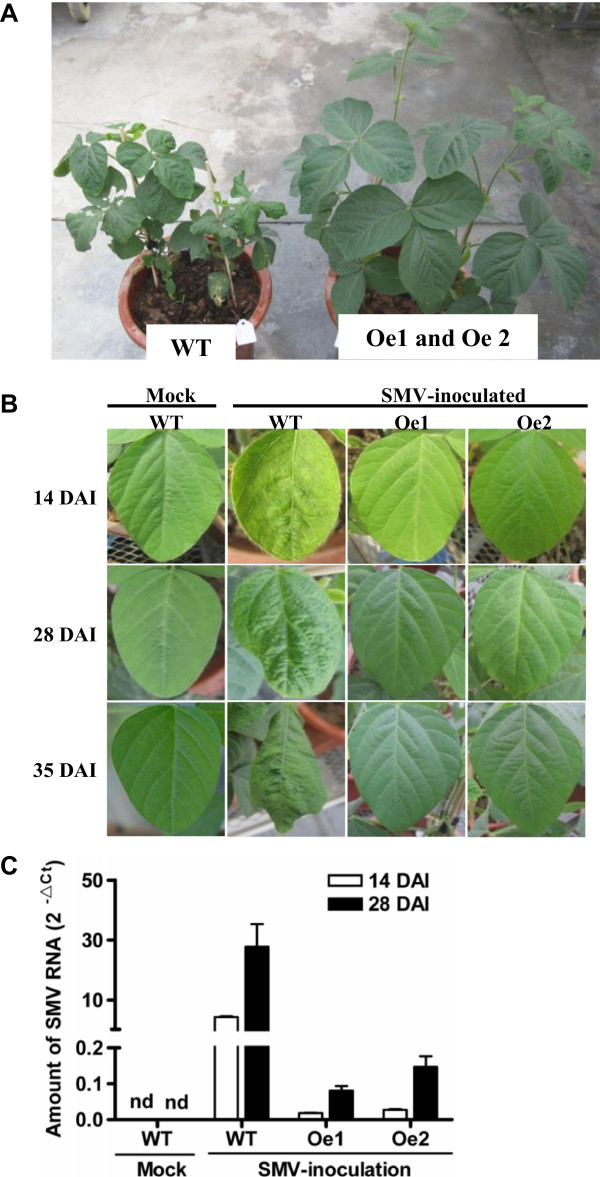
**Overexpression of *GmAKT2 *enhanced SMV resistance. (A)** SMV symptoms in the WT and GmAKT2 overexpression plants at 35 DAI. Ten-day-old soybean plants in low-K soil with unrolled unifoliate leaves were mechanically inoculated with SMV or buffer (Mock). **(B)** SMV symptoms on the newest trifoliate leaves of Williams 82 and *GmAKT2*-overexpressing transgenic lines Oe1 and Oe2 at 14, 28, and 35 DAI. **(C)** Amount of SMV RNA detected by qRT-PCR. The middle leaflets of the leaves of Williams 82, and *GmAKT2*-ovexpression lines Oe1 and Oe2 were sampled at 14 and 28 DAI to extract total RNA for quantitative RT-PCR analysis. Transcript levels were calculated using the formula 2^-ΔCt^ for the expression levels relative to *GmACTIN*. Data represent means of three biological replicates with error bars indicating SD.

To determine the effect of *GmAKT2* overexpression on SMV resistance, *GmAKT2*-Oe and WT plants were grown in pots with soil without the addition of K fertilizer in greenhouses. At least five plants were inoculated with SMV G7 or buffer in their unrolled unifoliate leaves at the VC stage [35] when the unifoliate leaves were fully expanded. Starting from 14 DAI, while severe mosaic symptoms were observed on the leaves of the WT plants, the *GmAKT2*-overexpression lines Oe1 and Oe2 showed no symptom of SMV infection (Figure 6A, 6B).

To confirm the results, qRT-PCR was used to determine SMV virus accumulation in these soybean plants. Compared to those in the Mock treatment, SMV inoculation significantly increased the amount of SMV virus in both WT and *GmAKT2*-Oe plants. At 14 DAI, the level of SMV RNA in WT plants were about 240 and 160 times greater than that in the *GmAKT2*- Oe1 and Oe2 lines, respectively (Figure 6C). With the increase of the infection process, at 28 DAI, the amount of SMV RNA in the WT was further increased. It was about 260 times as much as that of the average level of SMV RNA in *GmAKT2*-overexpression lines.

To verify whether the SMV resistance conferred by *GmAKT2* overexpression is also effective on the other SMV stain, we tested the resistance of the transgenic plants to SMV strain G3. Results showed that the transgenic lines *GmAKT2*-Oe1 and Oe2 were also resistant to the infection of G3. Overexpression of *GmAKT2* significantly reduced the amount of G3 RNA in plants (Additional file 1: Figure S2).

The increased virus resistance in the transgenic lines *GmAKT2*-Oe1 and Oe2 significantly increased their growth performance and yield when the plants inoculated SMV strain G7. As shown in Table 2, the SMV infection significantly reduced the plant height, numbers of node, pod, and seed, and the seed weight per plant of the WT soybean. In contrast, the infection of SMV virus slightly affected those agronomic traits. The difference in those traits between the infected plants and Mock was not significant.

**Table 2:** **Agronomic performance of Williams 82 and *GmAKT2*-overexpressing transgenic (Oe1 and Oe2) plants with or without SMV inoculation**

**Genotype**	**Mock**			**SMV Inoculation**		
	**WT**	**Oe1**	**Oe2**	**WT**	**Oe1**	**Oe2**
Plant height (cm)	62.4 ± 3.5^a^	63.3 ± 2.8^a^	64.9 ± 3.1^a^	48.4 ± 6.4^b^	62.3 ± 3.9^a^	62.7 ± 3.3^a^
Branch number	2.1 ± 0.6^a^	2.5 ± 0.5^a^	2.3 ± 0.7^a^	1.1 ± 0.7^b^	2.4 ± 0.5^a^	2.2 ± 0.4^a^
Node number	19.0 ± 1.6^a^	19.3 ± 1.1^a^	20.1 ± 1.0^a^	16.8 ± 0.9^b^	18.8 ± 0.6^a^	20.3 ± 0.8^a^
Pod number/plant	53.9 ± 7.4^a^	54.1 ± 7.0^a^	51.9 ± 6.5^a^	26.7 ± 7.1^b^	51.3 ± 8.5^a^	49.5 ± 9.4^a^
Seed number/plant	95.1 ± 12.4^a^	98.0 ± 8.9^a^	94.6 ± 10.2^a^	46.5 ± 12.8^b^	95.0 ± 16.1^a^	90.3 ± 14.3^a^
Seed weight (g)/plant	11.0 ± 1.0^ab^	11.5 ± 0.9^a^	11.0 ± 2.0^ab^	4.9 ± 1.4^c^	10.6 ± 2.1^ab^	10.0 ± 1.3^b^
100 seed weight (g)	11.7 ± 0.9^a^	11.8 ± 1.0^a^	11.5 ± 1.1^a^	10.5 ± 0.7^b^	11.1 ± 0.7^ab^	11.2 ± 0.8^ab^
Seed coat browning	No	No	No	Yes	No	No

Data collected from a field experiment in Anhui province, 2012. Data are given as means ± SD. Different letters in a row indicate significant differences (LSD, P <0.05).

## Discussion

*Arabidopsis* AKT2/3 is a rectifying K^+^ channel that regulates K^+^ loading in phloem in source tissues and the unloading in sink tissues [32-34,36]. In this study, the soybean *AtAKT2* orthologous gene, *GmAKT2* was overexpressed in soybean. The amino acid sequence similarity between AtAKT2 and GmAKT2 is 63% and they are phylogenetically classified in the same subcluster (Additional file 1: Figure S3). It is proposed that GmAKT2 functions similarly as AtAKT2/3 to regulate the K^+^ loading in phloem. Indeed, we observed that overexpression of *GmAKT2* enhanced K^+^ reallocation from the source (older leaves, first trifoliate = oldest leaf)) to the sink (younger leaves, 7^th^ trifoliate = youngest), which led to a significant increase in K^+^ concentrations in the latter (Figure 5A, 5B).

In addition to the enhancement of K^+^ transport, overexpression of *GmAKT2* on a SMV-susceptible genotype Williams 82 significantly retarded the development of SMV and resulted in higher resistance to SMV (Figure 6A, B, C). Plant resistance to viral disease can be achieved by inhibiting viral long-distance movement. In a typical virus infection process, virus moves from the source leaves to the sink leaves via the phloem transport system [37], similar to the direction of K^+^ movement. A mutation in *AtAKT2* was found to inhibit sugar loading into the phloem of Arabidopsis [28]. It is possible that overexpression of the soybean *AKT2* resulted in changes in phloem environment to be unfavorable to virus loading, movement and unloading. Identification of the factor(s) to link the K^+^ transportation and virus movement will help elucidate the mechanism of how the *GmAKT2* overexpression in soybean results in greater resistance to SMV.

Plant resistance to virus disease could also be achieved by inhibition of the viral multiplication. As expected, plant nutritional status affects plant health and, consequently, influences plant resistance to pathogen infection. In this study, the application of K^+^ fertilizer significantly reduced the SMV accumulation in the SMV-susceptible cultivar Williams 82. Thus, the sufficient K^+^ concentrations in soybean plant cells should be important for suppression of SMV spread. On the other hand, in the SMV-resistant *GmAKT2* overexpression plants, the increase of K^+^ concentrations only occurred in younger leaves, but not in the older leaves. The results suggest that maintenance sufficient K^+^ content in young leaves should be important for soybean resistance to virus infection.

## Conclusions

The soybean potassium channel gene, *GmAKT2* was significantly induced by SMV inoculation in the SMV highly-resistant genotype Rsmv1, but not in the highly-susceptible genotype Ssmv1. The role of *GmAKT2* on SMV resistance was evaluated in the study. Results showed that overexpression of *GmAKT2* on a SMV-susceptible genotype Williams 82 enhanced the reallocation of K^+^ in the soybean plants and increased K^+^ concentrations in young leaves. Furthermore, SMV development was significantly retarded in *GmAKT2*-overexpressing transgenic soybean plants following SMV inoculation. These data provide a novel route for initiating molecular breeding to improve SMV resistance in soybean, an important crop world wide.

## Methods

### Plant materials and growth conditions

The soybean cultivar Williams 82 was used as the recipient genotype for soybean transformation. Rsmv1 and Ssmv1 were Chinese germplasm highly-resistant or highly-susceptible to SMV, respectively (Dr. Yuming Wang, personal communication).

For qRT-PCR analysis of *GmAKT2* expression, soybean seeds were germinated in sterile sand for one week and transferred into modified half-strength Hoagland nutrient solution [2.5 mM KNO_3_, 2.5 mM Ca(NO_3_)_2_, 0.5 mM KH_2_PO_4_, 0.25 mM K_2_SO_4_, 1 mM MgSO_4_, 0.023 mM H_3_BO_3_, 4.57 μM MnCl_2_, 3.8 μM ZnSO_4_, 0.09 μM (NH_4_)_6_Mo_7_O_24_, 1.57 mM CuSO_4_, and 0.1 mM Fe-EDTA(Na)] [38] with aeration. Plants with the nutrient deficient treatments were planted in the hydroponic solution eliminating the corresponding nutrient for 7 days prior to sampling. Roots (R), unifoliate leaves (UL), trifoliate leaves (TL), stems (S), flowers (F), and pods (P) samples were collected on the 6-week-old plants grown hydroponically. Soybean plants were grown in growth chambers with a 12-hour photoperiod (1000 μmol photons m^-2^ sec^-1^) with temperature regimes of 30°C/22°C.

For soil experiments, soil with poor initial nutrient levels was collected. Nitrogen and phosphate fertilizers were applied to avoid nitrogen and phosphorus deficiency. The initial soil K^+^ level was 36.5 mg/kg and the K^+^ sufficient soil was 200 mg/kg by application of potassium sulfate. For the experiments to determine the effect of K supply on SMV incidence, 10 plants were included for each treatment. Three biological replications were employed.

For evaluation of agronomic traits, 10 WT plants (Williams 82) and 10 of each transgenic line were randomly sampled at the mature stage from the experimental station in Anhui province, China. To evaluate the effect of SMV infection on the agronomic traits, 12 WT and 12 of each transgenic line that either was Mock-treated or inoculated with SMV G7 strain at 10 day seedling stage in the greenhouse were harvested at mature stage. Plant height, branch number, node number, pod number, seed number, seed weight, and 100-seed weights were measured.

### Soybean mosaic virus inoculation and sampling

Soybean plants were grown in soil pots for 10 days prior to inoculation. The SMV G7 strain was used for inoculation. Inoculums were prepared by grinding infected soybean leaf tissues in 50 mM phosphate buffer, pH 7.0. The unrolled unifoliate leaves were inoculated by gently rubbing with the inoculums. Plants inoculated with buffer alone were used as Mock controls.

The middle leaflets of the all trifoliate leaves of the plants at 14 and 28 DAI for RNA extraction and qRT-PCR analysis were collected and mixed for RNA extraction. Three biological replicates were applied for inoculation and sampling.

### Construction of transgenic plants

The full-length cDNA of soybean *GmAKT2* (Glym08g20030.1) was amplified by RT-PCR and ligated into a TOPO vector (Invitrogen, Carlsbad, CA, USA). *GmAKT2* was cloned into the gateway plasmid pB7RWG2,0 (Invitrogen) under the control of the 35S promoter of cauliflower mosaic virus (CaMV). The resulting construct was introduced into the Agrobacterium strain EHA101. Transgenic soybean plants were generated using the *Agrobacterium tumefaciens*-mediated cotyledon node transformation system [39]. The Williams 82 genotype was used as the recipient of the transformation.

### Genomic DNA extraction and southern blot analysis

Soybean leaf genomic DNA was extracted using a modified CTAB method [40]. Thirty micrograms of genomic DNA from each sample was digested with *Hin*dIII that cuts once in the T-DNA region (Figure 4A). The digested DNA was separated on 1% agarose gels at 20 V for 20 hours followed by denaturation and neutralization. DNA samples were transferred onto nitrocellulose membranes by capillary action. Nucleic acids were fixed to the membranes by cross-linking with UV-light. A digoxin (DIG)-labeled *bar* probe was synthesized and detected with DIG-High Prime DNA Labeling and Detection Starter Kit I (Roche Applied Science, Indianapolis, IN, USA) according to the manufacturer’s instructions.

### RNA Extraction and quantitative real-time PCR

RNA was extracted using TRIzol Reagent (Invitrogen) according to the manufacturer’s instructions. DNaseI digestion was performed to ensure the absence of genomic DNA (Takara, Dalian, China).

First-strand cDNA was synthesized from total RNA using Moloney Murine Leukemia Virus Reverse Transcriptase (M-MLV RT, Promega, San Luis Obispo, CA, USA). qRT-PCR was performed using SYBR Green I on a Light Cycler 480 (Roche Applied Science, Indianapolis, IN, USA), according to the manufacturer’s instructions. The amplification program for SYBR Green I was performed as 94°C for 10 sec, 58°C for 10 sec, and 72°C for 10 sec. Triplicate quantitative assays were performed for each cDNA sample. *GmACTIN* was used as an internal control. Transcript levels were calculated using the formula 2^-ΔCt^ for relative expression or 2^-ΔΔCt^ for the expression levels relative to *GmACTIN*. Virus accumulation in soybean plants was estimated by qRT-PCR analysis of the SMV polyprotein gene in the SMV strain G7 or G3 genome. All primers used for RT-PCR are provided in Additional file 2: Table S1.

### Measurement of potassium concentration

Soybean plants were grown in pots containing K-sufficient or -starvation soil. The first through seventh trifoliate leaves of the 6-week-old plants were collected for ion concentration analysis. Plant materials were sampled and dried at 80°C for 3 days. Then, 50 mg of dry material was dissolved in 3 ml nitric acid and 2 ml H_2_O_2_ (30 %). The digested samples were diluted to a total volume of 50 ml with ultrapure water and transferred into new tubes and analyzed using a PerkinElmer ELAN® ICP-MS system.

## Competing interests

The authors declare that they have no competing interests.

## Authors’ contributions

HS and BL conceived and designed the research. LZ, HH, RL, and QH conducted the experiments and analyzed the data. LZ and HS wrote the manuscript. All authors read and approved the manuscript.

## Supplementary Material

Additional file 1: Figure S1 Sequence of *GmAKT2* full-length cDNA. **Figure S2.** Amount of SMV RNA. Ten-day-old soybean plants in low-K soil with unrolled unifoliate leaves were mechanically inoculated with SMV G7, G3 or buffer (Mock). Soybean trifoliate leaves were sampled at 14 and 28 DAI to extract total RNA for qRT-PCR analysis of SMV. Transcript levels were calculated using the formula 2^-ΔCt^ for the expression levels relative to *GmACTIN*. Data represent means of four biological replicates with error bars indicating SD. **Figure S3.** Phylogenetic analysis of soybean and *Arabidopsis Shaker* family proteins. This tree was obtained using the whole alignment and distance method. GmAKT2 and AtAKT2 formed a separate branch. The bar indicates the mean distance of 0.1 changes per amino acid residue. Click here for file

Additional file 2: Table S1 Sequences of primer pairs used for RT-PCR with the accession numbers of the genes. Click here for file
