# Supplementary material for: Overexpression of GmAKT2 potassium channel enhances resistance to soybean mosaic virus
Source: BMC Plant Biol. 2014 Jun 3;14:154. doi: 10.1186/1471-2229-14-154 (PMC4074861; doi:10.1186/1471-2229-14-154)
Supplement: Additional file 1: Figure S1 — Sequence of GmAKT2 full-length cDNA. Figure S2. Amount of SMV RNA. Ten-day-old soybean plants in low-K soil with unrolled unifoliate leaves were mechanically inoculated with SMV G7, G3 or buffer (Mock). Soybean trifoliate leaves were sampled at 14 and 28 DAI to extract total RNA for qRT-PCR analysis of SMV. Transcript levels were calculated using the formula 2-ΔCt for the expression levels relative to GmACTIN. Data represent means of four biological replicates with error bars indicating SD. Figure S3. Phylogenetic analysis of soybean and Arabidopsis Shaker family proteins. This tree was obtained using the whole alignment and distance method. GmAKT2 and AtAKT2 formed a separate branch. The bar indicates the mean distance of 0.1 changes per amino acid residue. [file 1471-2229-14-154-S1.pptx]

## Slide 1
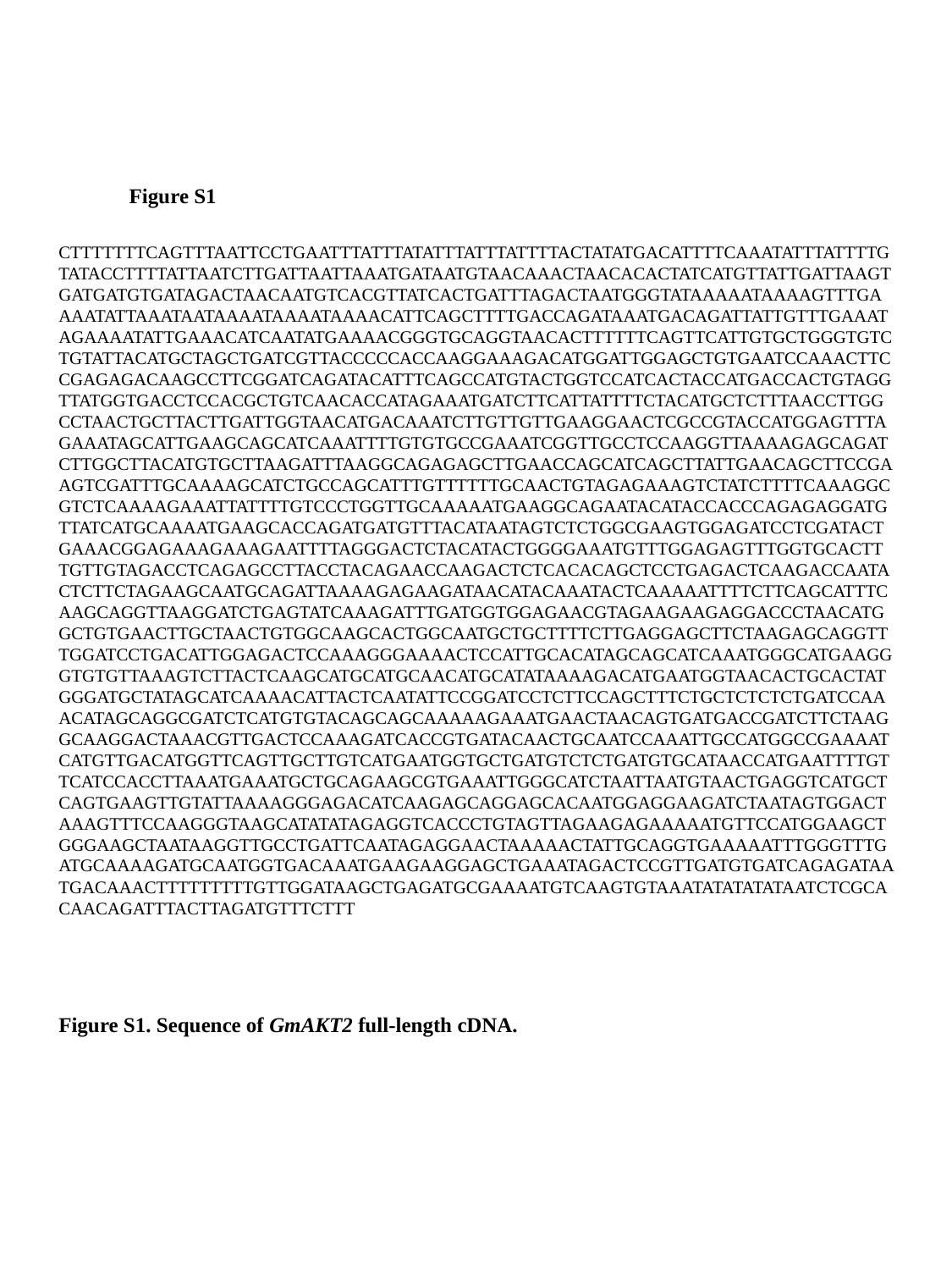

Figure S1
CTTTTTTTCAGTTTAATTCCTGAATTTATTTATATTTATTTATTTTACTATATGACATTTTCAAATATTTATTTTGTATACCTTTTATTAATCTTGATTAATTAAATGATAATGTAACAAACTAACACACTATCATGTTATTGATTAAGTGATGATGTGATAGACTAACAATGTCACGTTATCACTGATTTAGACTAATGGGTATAAAAATAAAAGTTTGAAAATATTAAATAATAAAATAAAATAAAACATTCAGCTTTTGACCAGATAAATGACAGATTATTGTTTGAAATAGAAAATATTGAAACATCAATATGAAAACGGGTGCAGGTAACACTTTTTTCAGTTCATTGTGCTGGGTGTCTGTATTACATGCTAGCTGATCGTTACCCCCACCAAGGAAAGACATGGATTGGAGCTGTGAATCCAAACTTCCGAGAGACAAGCCTTCGGATCAGATACATTTCAGCCATGTACTGGTCCATCACTACCATGACCACTGTAGGTTATGGTGACCTCCACGCTGTCAACACCATAGAAATGATCTTCATTATTTTCTACATGCTCTTTAACCTTGGCCTAACTGCTTACTTGATTGGTAACATGACAAATCTTGTTGTTGAAGGAACTCGCCGTACCATGGAGTTTAGAAATAGCATTGAAGCAGCATCAAATTTTGTGTGCCGAAATCGGTTGCCTCCAAGGTTAAAAGAGCAGATCTTGGCTTACATGTGCTTAAGATTTAAGGCAGAGAGCTTGAACCAGCATCAGCTTATTGAACAGCTTCCGAAGTCGATTTGCAAAAGCATCTGCCAGCATTTGTTTTTTGCAACTGTAGAGAAAGTCTATCTTTTCAAAGGCGTCTCAAAAGAAATTATTTTGTCCCTGGTTGCAAAAATGAAGGCAGAATACATACCACCCAGAGAGGATGTTATCATGCAAAATGAAGCACCAGATGATGTTTACATAATAGTCTCTGGCGAAGTGGAGATCCTCGATACTGAAACGGAGAAAGAAAGAATTTTAGGGACTCTACATACTGGGGAAATGTTTGGAGAGTTTGGTGCACTTTGTTGTAGACCTCAGAGCCTTACCTACAGAACCAAGACTCTCACACAGCTCCTGAGACTCAAGACCAATACTCTTCTAGAAGCAATGCAGATTAAAAGAGAAGATAACATACAAATACTCAAAAATTTTCTTCAGCATTTCAAGCAGGTTAAGGATCTGAGTATCAAAGATTTGATGGTGGAGAACGTAGAAGAAGAGGACCCTAACATGGCTGTGAACTTGCTAACTGTGGCAAGCACTGGCAATGCTGCTTTTCTTGAGGAGCTTCTAAGAGCAGGTTTGGATCCTGACATTGGAGACTCCAAAGGGAAAACTCCATTGCACATAGCAGCATCAAATGGGCATGAAGGGTGTGTTAAAGTCTTACTCAAGCATGCATGCAACATGCATATAAAAGACATGAATGGTAACACTGCACTATGGGATGCTATAGCATCAAAACATTACTCAATATTCCGGATCCTCTTCCAGCTTTCTGCTCTCTCTGATCCAAACATAGCAGGCGATCTCATGTGTACAGCAGCAAAAAGAAATGAACTAACAGTGATGACCGATCTTCTAAGGCAAGGACTAAACGTTGACTCCAAAGATCACCGTGATACAACTGCAATCCAAATTGCCATGGCCGAAAATCATGTTGACATGGTTCAGTTGCTTGTCATGAATGGTGCTGATGTCTCTGATGTGCATAACCATGAATTTTGTTCATCCACCTTAAATGAAATGCTGCAGAAGCGTGAAATTGGGCATCTAATTAATGTAACTGAGGTCATGCTCAGTGAAGTTGTATTAAAAGGGAGACATCAAGAGCAGGAGCACAATGGAGGAAGATCTAATAGTGGACTAAAGTTTCCAAGGGTAAGCATATATAGAGGTCACCCTGTAGTTAGAAGAGAAAAATGTTCCATGGAAGCTGGGAAGCTAATAAGGTTGCCTGATTCAATAGAGGAACTAAAAACTATTGCAGGTGAAAAATTTGGGTTTGATGCAAAAGATGCAATGGTGACAAATGAAGAAGGAGCTGAAATAGACTCCGTTGATGTGATCAGAGATAATGACAAACTTTTTTTTTGTTGGATAAGCTGAGATGCGAAAATGTCAAGTGTAAATATATATATAATCTCGCACAACAGATTTACTTAGATGTTTCTTT
Figure S1. Sequence of GmAKT2 full-length cDNA.

## Slide 2
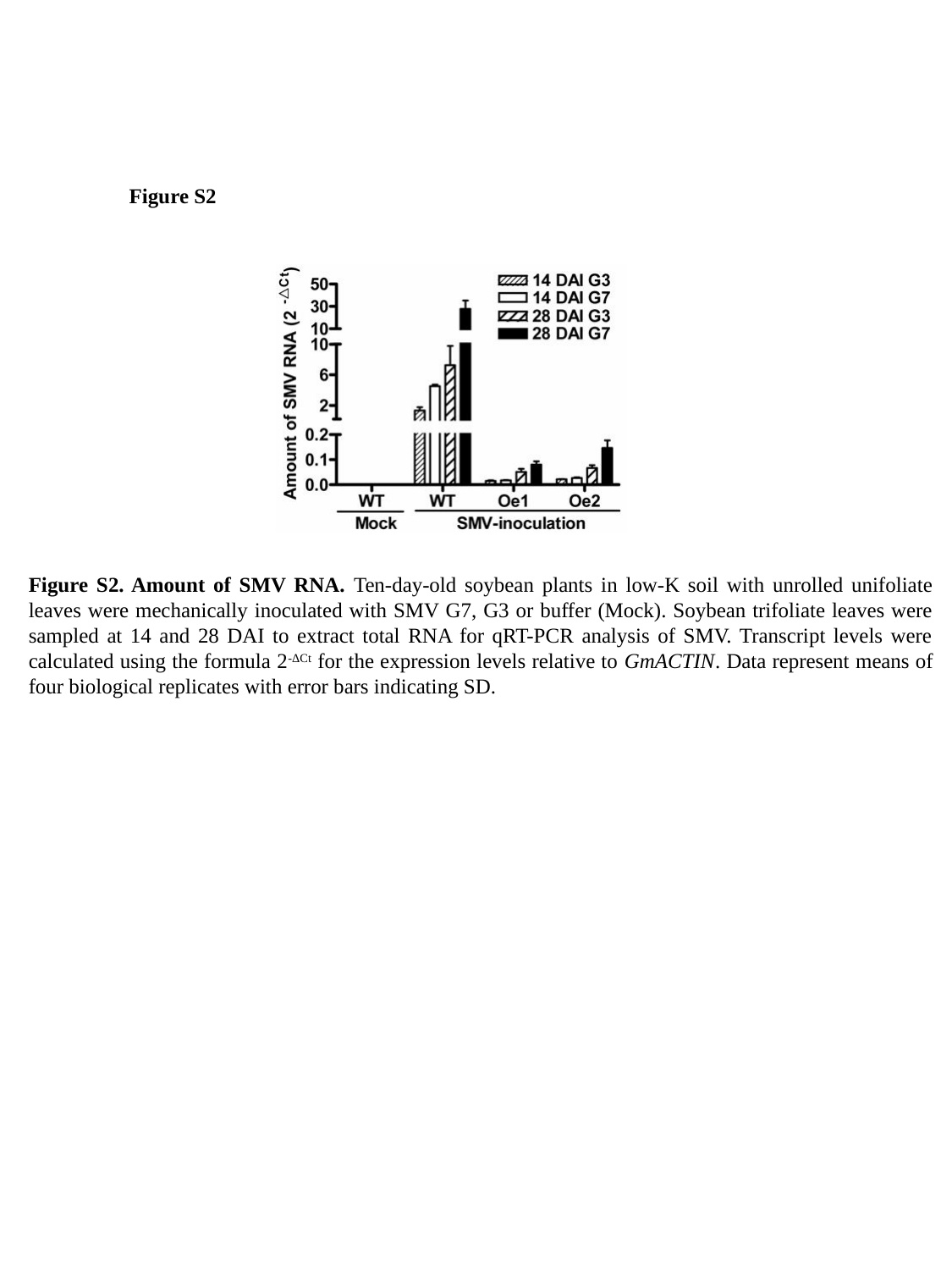

Figure S2
Figure S2. Amount of SMV RNA. Ten-day-old soybean plants in low-K soil with unrolled unifoliate leaves were mechanically inoculated with SMV G7, G3 or buffer (Mock). Soybean trifoliate leaves were sampled at 14 and 28 DAI to extract total RNA for qRT-PCR analysis of SMV. Transcript levels were calculated using the formula 2-ΔCt for the expression levels relative to GmACTIN. Data represent means of four biological replicates with error bars indicating SD.

## Slide 3
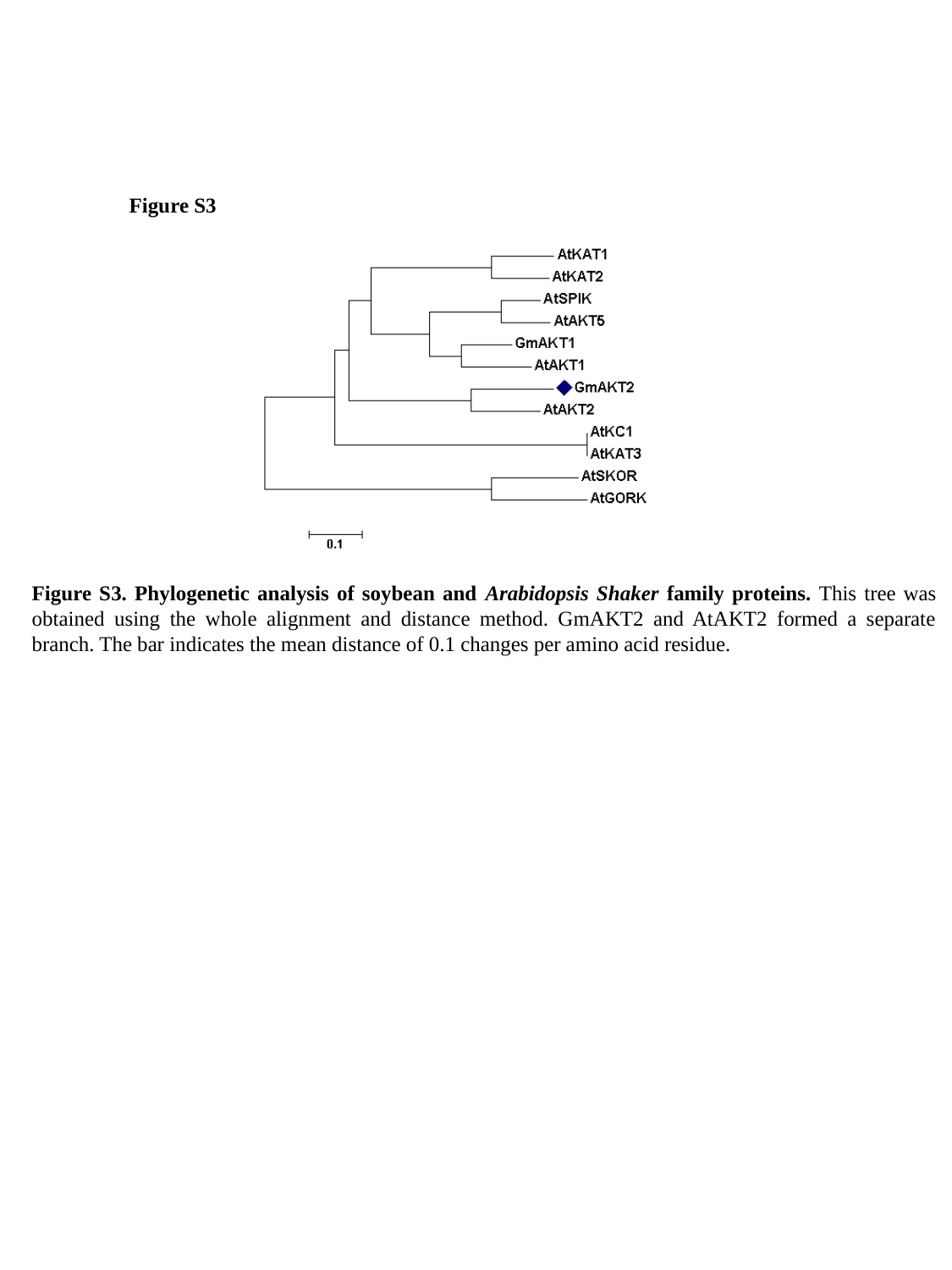

Figure S3
Figure S3. Phylogenetic analysis of soybean and Arabidopsis Shaker family proteins. This tree was obtained using the whole alignment and distance method. GmAKT2 and AtAKT2 formed a separate branch. The bar indicates the mean distance of 0.1 changes per amino acid residue.
